# Development of breast tissue–mimicking electrical and acoustic phantoms for magneto‐acoustic electrical tomography

**DOI:** 10.1111/nyas.15338

**Published:** 2025-04-15

**Authors:** Reyhan Zengin, Nevzat Güneri Gençer

**Affiliations:** ^1^ Department of Biomedical Engineering Inonu University Malatya Turkey; ^2^ Department of Electrical and Electronics Engineering Middle East Technical University Ankara Turkey

**Keywords:** acoustic, electrical properties, magneto‐acousto electrical tomography, tissue‐mimicking breast phantoms

## Abstract

Magneto‐acousto electrical tomography (MAET) is a novel medical imaging technique that relies on the difference in electrical properties between healthy and tumor tissues. To facilitate MAET experiments, this study proposes a comprehensive procedure for developing, characterizing, and preserving realistic breast tissue–mimicking phantoms. We developed nontoxic and inexpensive phantoms using sodium alginate, graphite powder, agar, propanediol, aluminum powder, glycine, and deionized water. The dielectric (conductivity and permittivity) and acoustic (speed of sound) properties of phantoms (breast fat, breast gland, and tumor) within the 1–8 MHz frequency range were measured to ensure their suitability for MAET experiments. In conclusion, this study presents a detailed methodology for the preparation, characterization, and preservation of realistic breast tissue–mimicking phantoms tailored for MAET experiments.

## Graphical abstract

In this study, breast tissue–mimicking phantoms were developed to simulate the electrical conductivity, permittivity, and speed of sound of breast fat, glandular tissue, and tumors within the 1–8 MHz range for magneto‐acousto electrical tomography (MAET) experiments. The electrical and acoustic properties of these phantoms were measured and compared with real tissue values, highlighting their potential for tumor detection. These phantoms provide a valuable tool for improving the accuracy of non‐invasive imaging techniques, particularly MAET, in diagnostic settings.

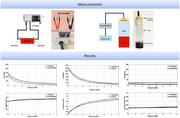

## INTRODUCTION

Magneto‐acousto electrical tomography (MAET) is a technique used for imaging the electrical properties of biological tissues.^1^, ^2^, ^3^, ^4^, ^5^, ^6^, ^7^, ^8^, ^9^ Ultrasound and electromagnetic fields are combined to generate current distribution in the body via MAET. Developing phantoms that mimic the different (acoustic, electrical, mechanical) properties of tissues is crucial for validating new imaging methods. In this study, we aimed to develop phantoms that accurately simulate breast tissue's acoustic and electrical properties to utilize them to assess the efficacy of MAET. This study was carried out by accurately calculating the electrical and acoustic properties of phantoms that closely mimic the characteristics of breast tissues, specifically within the low‐frequency range of 1–8 MHz, marking a significant development in this field.

Tissue‐mimicking (TM) phantoms are used in ultrasound systems to assess the acoustic characteristics of tissues, including attenuation, absorption, scattering, and propagation velocity. Several studies have been conducted to investigate the properties of these phantoms.^10^, ^11^, ^12^, ^13^, ^14^, ^15^, ^16^, ^17^, ^18^, ^19^, ^20^, ^21^, ^22^ Insana et al. used water‐based gelatin mixed with graphite powder to determine the ultrasonic scattering properties of TM phantoms^10^ and, in a separate study, investigated the ultrasonic attenuation of these phantoms.^11^ Madsen et al. researched the velocities of shear waves in soft tissues and their attenuation coefficients using silicone rubber and gel to mimic tissue‐like substances and examined fresh bovine tissues at 2–14 MHz.^12^ Chin et al. prepared a thermal model of an ultrasonic TM muscle‐like phantom using an array of copper–constantan thermocouples to test time‐dependent temperature distributions with a 525 kHz transducer.^13^ Wu et al. evaluated the temperature increase generated by focused and unfocused transducers at 1.0 and 3.5 MHz frequencies with homogeneous phantoms made of unflavored gelatin, boiled degassed water, olive oil, and castor oil, designed to mimic thermal and acoustic properties.^14^ Zhou et al. developed four agar phantoms to estimate ultrasonic attenuation through the difference ratio correction method with a 3.5 MHz transducer.^23^ Dabrowski et al. compared images obtained from X‐ray, angiography, computed tomography, and 3D B‐mode ultrasound using a natural human vessel placed in an agar‐filled acrylic box.^24^ Madsen et al. proposed a new TM material for ultrasound consisting of evaporated milk as the primary absorption component and demonstrated low backscatter and a propagation speed of 1540 m/s.^25^ Wu et al. measured the acoustic properties of tofu and suggested its use as a TM material for ultrasonic applications.^15^ Madsen et al. produced five TM agar/gelatin materials for heterogeneous elastography phantoms.^16^ Inglis et al. constructed an anthropomorphic phantom of the esophagus for endoscopic ultrasound.^17^ Zell et al. characterized the acoustic properties of four materials (agar, silicone, polyvinyl alcohol [PVA] gel, and polyacrylamide [PAA] gel) for tissue phantoms used in photoacoustic and ultrasound imaging.^18^ Cannon et al. developed novel agar‐based TM materials and measured their acoustic properties.^19^ Vieira et al. investigated paraffin‐gel waxes as new soft TM materials for ultrasound‐guided breast biopsy training,^20^ whereas Ng and Lin examined the tunability of acoustic and mechanical behaviors in breast TM materials.^21^ Filippou and Damianou developed agar‐based phantoms with silica and evaluated their acoustic properties via pulse‐echo and transmission techniques.^22^ Braunstein et al. developed TM phantoms made with PVA hydrogels to evaluate their therapeutic potential, taking advantage of the thermal and mechanical effects induced during ultrasound use.^26^

TM phantoms are crucial for advancing diagnostic technologies. They are designed to mimic biological tissues’ electrical and often mechanical properties, providing a reliable platform for testing and improving various imaging modalities. Typically, these phantoms incorporate saline, agar, gelatin, and vegetables to closely mimic human tissues’ properties. Studies in this field include the development of gellan gum‐based phantoms by Chen et al., which offer precise control over the elastic modulus and thermal and electrical conductivity between 100 Hz and 1 MHz.^27^ Similarly, Li et al. have formulated a breast TM phantom suitable for microwave and ultrasound imaging, employing a mixture of coconut oil, canola oil, agar, glass beads, and polyvinylpyrrolidone to simulate different tissue types, including skin, fat, fibroglandular, and tumor tissues, between 200 MHz and 6 GHz.^28^ Moreover, Di Meo et al. have contributed by examining the dielectric and mechanical characteristics of breast phantoms comprising sunflower oil, deionized water, dishwashing liquid, and gelatin, with a focus on electrical property measurements across a broad frequency spectrum (500 MHz to 14 GHz).^29^

Our research builds upon these studies by uniquely simulating breast tissue's conductivity and permittivity properties, utilizing chemical materials over a 1–8 MHz frequency range. A distinctive feature of our work is the matched speed of sound in our phantoms to that in actual breast tissues, significantly enhancing model accuracy. This aspect sets our study apart from other works, such as the phantom proposed by Sirtoli et al.,^30^ which, while innovative in mimicking specific electrical parameters for cancer detection, does not address both permittivity and speed of sound, elements critical for a tissue simulation.

Various studies have employed a technique for preparing well‐defined phantoms via discrete electronic components.^31^, ^32^, ^33^, ^34^, ^35^ This method produces phantoms with well‐defined values but lacks intricate interactions among electrodes in distributed models or realistic tissues. Sodium chloride solutions mixed with a small percentage of agar or gelatin by weight have been acknowledged as excellent electrical impedance tomography (EIT) phantoms, possessing several functional properties.^36^, ^37^, ^38^ However, agar/saline phantoms fail to mimic the permittivity of realistic tissue. Attempts to address this limitation have included incorporating biological materials with cellular structures, such as bananas or cucumbers, which contribute substantially to the permittivity of the solid structure.^39^, ^40^, ^41^ Despite challenges in controlling or reproducing their electrical properties and perishability, other substances such as TX 151^42^, ^43^ and PAA^39^, ^44^ have been proposed for use in EIT and higher frequencies. Graphite has proven valuable in simulating the conductivity and permittivity of phantoms at microwave frequencies and is also suitable for constructing phantoms for ultrasound imaging.^45^, ^46^, ^47^, ^48^ Agar fails to generate the characteristic speckle in ultrasound images when used alone. However, incorporating graphite introduces this feature, resulting in more lifelike ultrasound images, making graphite an appealing medium for constructing phantoms in dual‐mode imaging systems for EIT and ultrasound.^49^

MAET has demonstrated the ability to detect small tumors based on changes in electrical conductivity and acoustic properties. Han et al.^50^ provided initial evidence of this by using a 6.0 cm × 1.2 cm polystyrene block immersed in saline, which demonstrated the basic feasibility of Hall effect imaging and the ability to detect small conductivity contrasts. Xu et al.^2^ further validated this technique with a 1.8‐cm thick slab immersed in insulating oil, confirming that even small conductivity differences could be detected. Haider et al.^3^ used a 0.8‐cm thick slab to demonstrate MAET's capability to detect small electrical conductivity differences in thin materials. Zeng et al.^5^ modeled a sample with dimensions of 100 mm length, 1 mm width, and 50 mm height, showing that MAET could resolve small conductivity variations, including tumors as small as a few millimeters. Grasland‐Mongrain et al.^6^ demonstrated MAET's ability to differentiate between muscle and fat tissue using an 8‐cm wide beef muscle tissue phantom, showing its sensitivity to small conductivity contrasts. Guo et al.^8^ confirmed the technique's efficacy using a 4 cm × 1 cm cube, detecting small conductivity changes even in smaller phantom sizes. Kunyansky et al.^51^ and Zhou et al.^52^ provided further support for MAET's ability to resolve small conductivity differences, with Zhou's study detecting breast tumors with conductivity differences as low as 0.1 S/m. Kunyansky et al.^51^ used a 28‐mm diameter lard cylinder to differentiate between conductive and nonconductive materials, supporting MAET's ability to resolve small conductivity differences and detect small tumors. Zhou et al.^52^ conducted an experiment with a 6 cm × 5 cm tumor phantom to simulate a breast tumor, demonstrating that MAET could detect small tumors with good accuracy, even when conductivity differences were as low as 0.1 S/m. Yu et al.^53^ conducted a study using a 50‐mm‐side‐length, three‐layer gel phantom with layers of varying conductivity (1, 0.05, and 1 S/m), where MAET successfully detected the conductivity differences between the layers, demonstrating its sensitivity to small contrasts. Li et al.^54^ employed a gel phantom with a 20‐mm thick first layer and a 10‐mm thick second layer, simulating a liver tumor with a 25‐mm diameter, and showed that MAET could differentiate small conductivity changes, highlighting its effectiveness for tumor detection. Sun et al.^55^ used a 50 mm × 60 mm set of silver‐plated electrodes in conjunction with a 50 mm gel phantom, demonstrating that MAET could detect small conductivity variations across different materials effectively. Li et al.^56^ simulated tumors with 8 mm × 5 mm dimensions in a breast tissue phantom, and the results confirmed that MAET could detect even small tumors, underscoring its high sensitivity to small conductivity contrasts. Liu et al.^57^ utilized a 1 cm × 1 cm × 1 cm cube to simulate uniform tissue with varying conductivity, showing that MAET could detect small conductivity differences and is suitable for tumor detection in small‐scale models. Together, these studies demonstrate MAET's high sensitivity and reliability in detecting tumors and small conductivity contrasts.

This study aims to develop phantoms using agar, sodium alginate, agar, propanediol, glycine, aluminum powder, and graphite powder to mimic breast tissues’ electrical and acoustic characteristics at low frequencies. The acoustic and electrical properties of the phantoms are compared to the values reported in the previous studies. Typically, investigations on phantoms simulating breast tissue have focused primarily on their electrical properties at high frequencies. However, our study precisely mimics electrical conductivity and dielectric constant values ranging from 1 to 8 MHz, which align with the operating frequencies of ultrasound imaging used for diagnostic purposes. The resulting phantoms will be employed in magnetic measurements for the MAET experiments.

In this paper, the Materials and Methods section details the preparation of phantoms and outlines the measurement setup for electrical properties and speed of sound. The Results section presents the findings on the electrical conductivity, dielectric constant, and speed of sound for the prepared phantoms. The last two sections comprise the discussion and conclusions drawn from the study's results.

## MATERIALS AND METHODS

### Phantom preparation

Phantoms can be used repeatedly in place of tissues in experimental studies by mimicking their mechanical, structural, acoustic, and dielectric properties. Phantoms with well‐known geometries and material structures are used to develop medical imaging systems. In this study, TM breast phantoms were developed to mimic ultrasonic and electrical properties. During phantom production, the acoustic (sound velocity) and electrical properties (conductivity and dielectric coefficient) were carefully considered.

We developed three phantoms to mimic breast tissue: the breast gland, breast fat, and tumor. The components used to develop each phantom included sodium alginate (a gelling agent), distilled water (a base material), agar (used for the speed of sound), propanediol, glycine, aluminum powder, and graphite powder (used for conductivity and permittivity). These phantoms were prepared via the following steps: (1) We added 75 mL of distilled water and 2 g of sodium alginate. We stirred the mixture with a magnetic stirrer for approximately 24 h (without heat). Sodium alginate, derived from marine brown algae cell walls, typically comprises approximately 30−60% alginic acid. This conversion to sodium alginate enhances water solubility, facilitating easier extraction processes.^58^ (2) Next, to ensure better mixing of the sodium alginate, we heated this homogeneous mixture to 80 degrees Celsius using a magnetic stirrer and heater. (3) Once the mixture cooled to 55–60 degrees Celsius, agar, propanediol, glycine, aluminum powder, graphite powder, and an antifungal agent were added. (4) To prevent evaporation, we covered the mixing bowl with aluminum foil and allowed it to stand at room temperature before refrigerating it for approximately 12 hours.

In this study, we mimicked the electrical properties of the tumor phantom as blood due to the high vascularity of tumors, which significantly influences their electrical properties. Blood plays a key role in determining the overall characteristics of tumor tissues, as highlighted by Egeblad et al.,^59^ who emphasized the importance of the vascular network within tumors. Table 1 lists the materials used for each phantom and their respective quantities. As shown in Table 1, modifying the concentrations of the other materials allows for the development of phantoms that better represent different tumor types and pathological conditions.

**TABLE 1:** The materials used for the phantoms and their amounts.

Material	Breast fat	Breast gland	Tumor
Sodium alginate (g)	2	2	2
Agar (g)	0.1	0.4	0.7
Graphite powder (g)	0.1	0.4	0.7
Propanediol (mL)	60	−	−
Aluminum powder (g)	−	8	12
Glycine (g)	−	0.5	1

Initially, we used gelatin to solidify the phantoms. However, a significant amount of gelatin was required to achieve a homogeneous dispersion of the phantom materials. We began using sodium alginate in all phantoms as a more practical alternative. This decision was based on the understanding that sodium alginate facilitates the solidification of the phantom and ensures a more uniform dispersion of the graphite powder. Sodium alginate is a well‐known hydrogel that is capable of absorbing water. Many biomedical applications utilize it extensively because of its biocompatibility, minimal toxicity, relatively affordable price, and gentle gelation ability.^60^ We used propanediol, which has a low dielectric constant among the prepared phantoms, to mimic the dielectric constant of fat tissue. In contrast, we used aluminum powder and glycine instead of propanediol to mimic the high dielectric constant of the breast gland and tumor phantoms.

The implementation of a range of precautions is crucial to maintain the durability and prevent fungal contamination of prepared phantoms. Ensuring the sterilization of all materials and equipment used in phantom preparation is essential for maintaining a safe and hygienic environment. This can be achieved through autoclaving, chemical disinfection, or irradiation.^61^ The addition of antifungal agents to phantom formulations can help prevent fungal growth. These agents may include fungicides or preservatives to inhibit fungal growth.^62^ However, it is crucial to consider the compatibility of these agents with phantom materials and their intended use. Our study used potassium sorbate, with nearly 1 mg for each phantom, to prevent fungal growth and contamination. Potassium sorbate is the most extensively utilized food preservative because it is classified as a generally recognized as safe additive.^63^ When added to the phantom formulation, potassium sorbate can inhibit the growth and reproduction of fungi, yeasts, and molds, reducing the risk of fungal contamination. In our experiment, however, mold growth was observed within 3 days despite refrigeration. While we did not conduct dedicated antibacterial testing in this study, literature findings suggest that potassium sorbate may have some inhibitory effects on bacterial growth. For example, at certain concentrations (e.g., 0.3%), it can also inhibit the growth of specific bacterial species, such as Salmonella.^64^

Stability measurements were conducted to evaluate the preservation of the phantom's electrical and acoustic properties over time. The results indicated that these properties remained stable for at least 15 days when stored in a refrigerator. While the overall structural integrity of the phantom was maintained for more than 2 months, the optimal preservation period depends on the specific application. If the system is prepared and used immediately, a shorter preservation time may be sufficient, whereas, for experiments requiring prolonged storage, the phantoms can be maintained for an extended duration under controlled conditions.

The phantom developed in this study specifically represents water‐content tumors. Malignant breast tumors generally exhibit higher water content than fatty tissues, which significantly influences their electrical properties. Surowiec et al. reported that at lower frequencies (1 MHz), the conductivity of malignant tissues ranges between 2 and 4 mS/cm (0.2–0.4 S/m), increasing to approximately 4–7 mS/cm (0.4–0.7 S/m) at higher frequencies (nearly 8 MHz).^65^ These values align with the characteristics of water‐rich tumors, supporting the rationale behind our phantom design.

B‐mode ultrasound imaging is typically performed using frequencies ranging from 1 to 15 MHz, with breast imaging specifically utilizing higher ultrasound frequencies in the range of 5–15 MHz.^66^ Additionally, medical ultrasound is widely used as a first‐line imaging modality due to its cost‐effectiveness and lack of ionizing radiation. Typical diagnostic ultrasound frequencies range from 1 to 20 MHz.^67^ Traditional medical ultrasound is primarily employed for diagnostic purposes; however, its therapeutic applications are increasingly gaining attention, further broadening its clinical significance. Given this, our study's focus on the 1–8 MHz range aligns well with diagnostic ultrasound applications for breast tissue.

### Electrical properties measurement setup

In this study, we measured the phantoms’ electrical conductivity and dielectric constants via a Hioki IM3536 inductance, capacitance, and resistance (LCR) meter with an operating frequency range of 4 kHz to 8 MHz.^68^ This LCR meter excites the phantoms using an AC constant current with an amplitude of 10 mA.

The resistance values (*R*) and conductivity values (*σ*) were calculated using the following formulas:

R=ρl/A

(1) σ=1/ρ,

where *l* is the distance between the plates, *A* is the area of the plates, and *ρ* is the resistivity.

**FIGURE 1:**
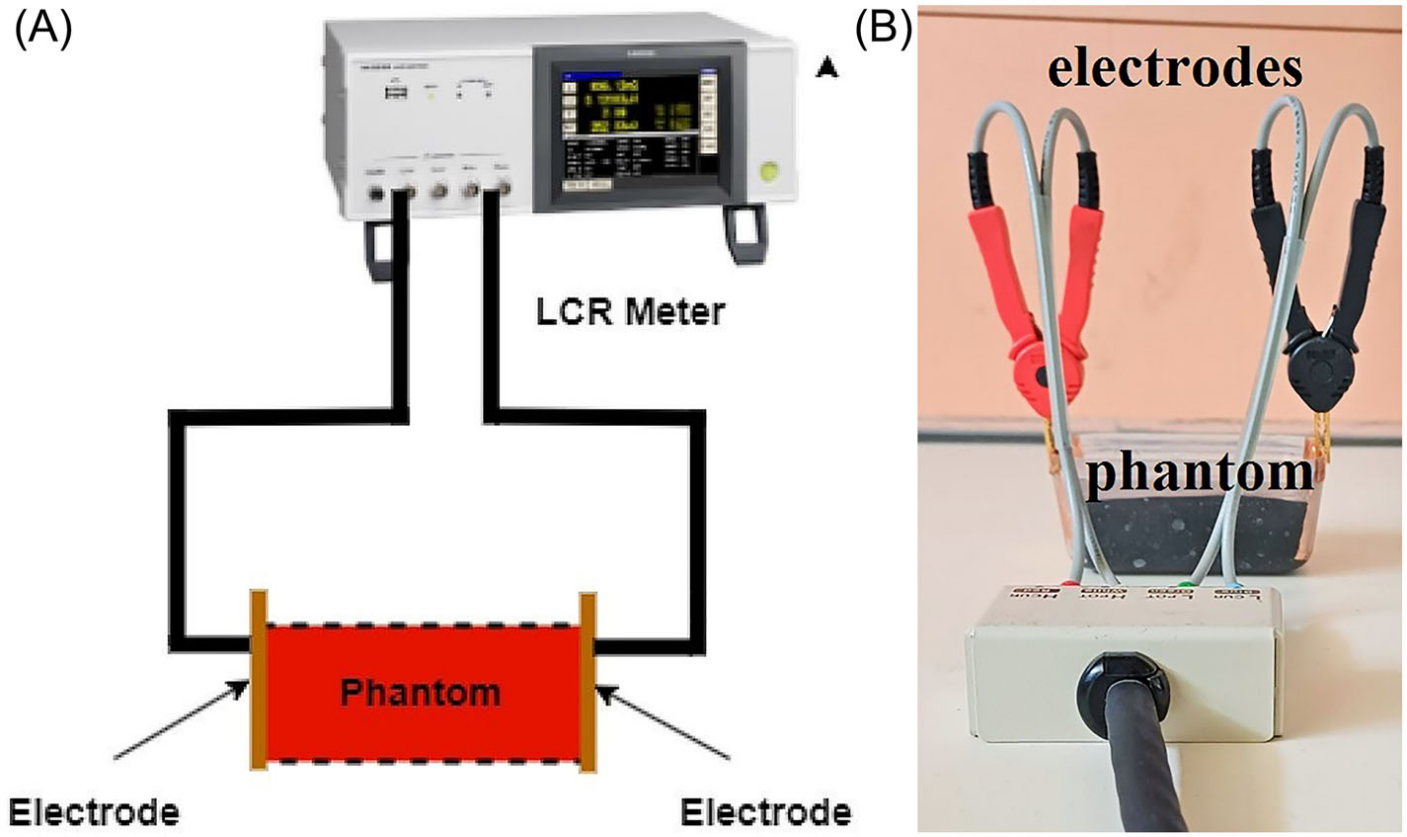
(A) Schematic and (B) photograph of the dielectric property measurement setup.

To measure the dielectric constant (permittivity) values of the prepared phantoms, the parallel‐plate method (capacitor method) is employed. This method uses parallel‐plate capacitors as the sample holder, and the phantom is positioned between these plates. An impedance analyzer or LCR meter is used to carry out the measurement, and the measurements are typically performed at low frequencies, usually below 1 GHz. The phantom is excited by an AC source, and the voltage across the sample is monitored. By determining the sample size and measuring the capacitor value, the dielectric constant of the sample can be calculated. The formula for calculating the dielectric constant of the prepared phantoms is given as follows:

(2) $$\in =\frac{Cd}{\in_{0}A},$$

where *ϵ*_0_ = 8.85 *×* 10*^−^* ^14^ F/cm (the permittivity of free space), *d* represents the distance between plates, *A* is the area of the plates, and *C* is the measured capacitor value. A schematic diagram of the measurement setup is shown in Figure 1A, and a photograph of the setup used to determine the dielectric properties is presented in Figure 1B.

### Speed of the sound measurement setup

Sound speed is a crucial characteristic of both solids and liquids. In ultrasonic imaging, which employs the pulse‐echo method, the sound speed is utilized to pinpoint interface locations. In biological tissue, an approximate speed of 1540 m/s is typically assumed,^69^ although this value can vary depending on the tissue type. Various methods exist for measuring sound speed, employing different techniques. Pellam and Galt^70^ introduced a method based on transmitting a pulse through a sample and detecting its reflection. Another commonly used method involves measuring the time‐of‐flight to and from a reflector with and without the sample,^71^ typically requiring a large bath for suspension. To address issues with echo detection from sample surfaces, several methods use two different coupling fluids for solids.^72^ Ophir and Lin^73^ developed a method for biological samples of unknown thickness, where samples are exposed to receiving hydrophones at known distances. These techniques necessitate setups integrating oscilloscopes, ultrasonic transducers, function generators, amplifiers, and plate reflectors.

**FIGURE 2:**
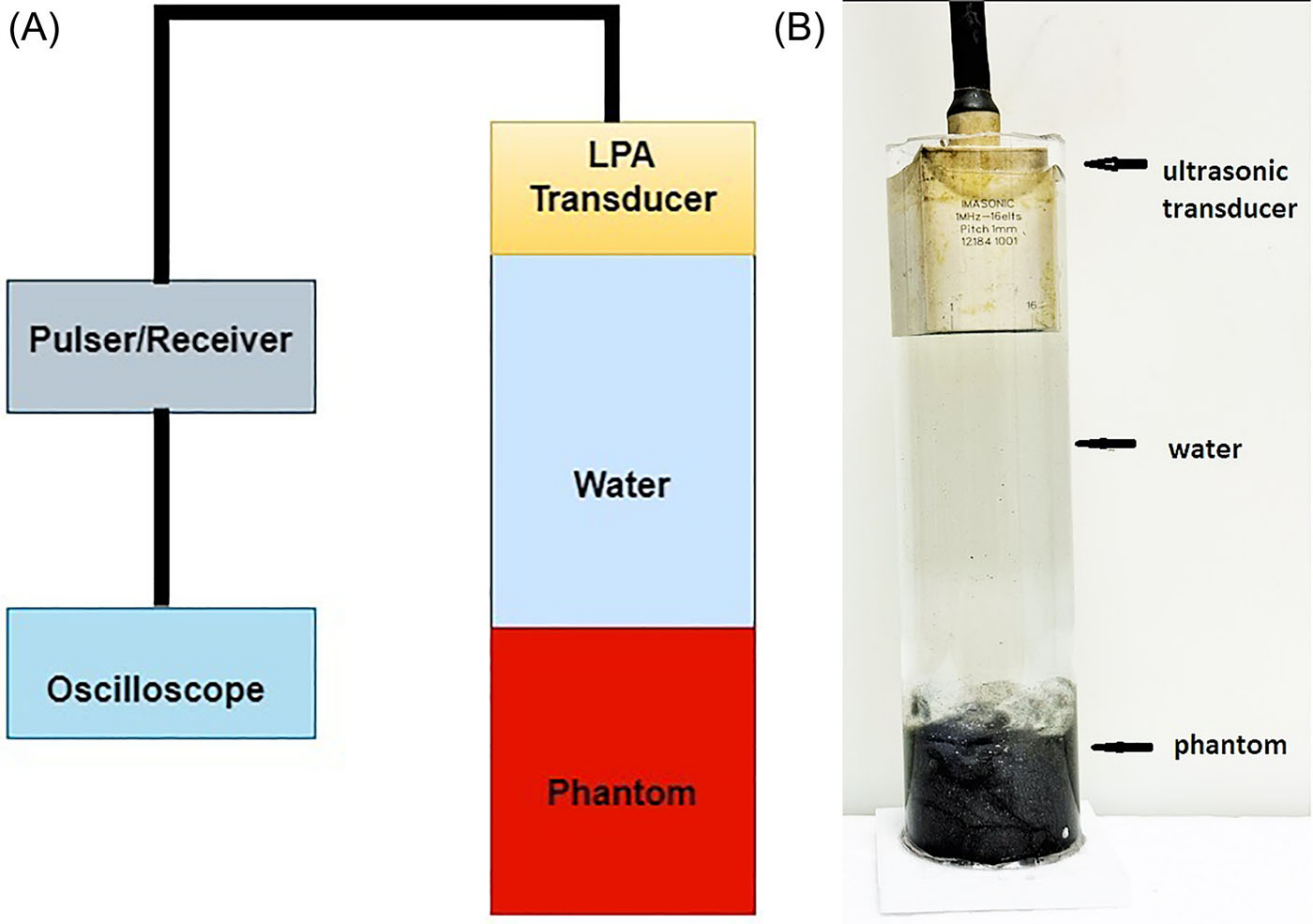
(A) Schematic and (B) photograph (right) of the speed of the sound measurement setup.

In this study, to measure the speed of sound in the phantoms, a 16‐element 1 MHz linear phased array (LPA) transducer (Imasonic) was employed, and a schematic and photograph of the setup are depicted in Figure 2A and B, respectively. The phantom is positioned in a glass tube and then filled with water. The LPA transducer is immersed in water and driven at a frequency of 1 MHz using 5 square waves of 100 *V_pp_*. The signal acquired in the amplitude mode of the transducer is observed on the oscilloscope.

## RESULTS

### Electrical conductivity and dielectric constant results of the phantoms

**FIGURE 3:**
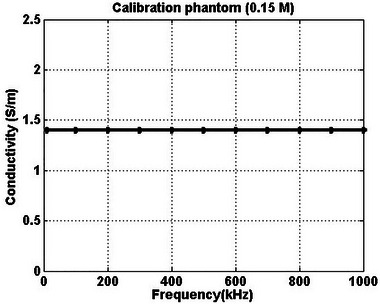
Electrical conductivity values of 0.15 M saline solution between 10 kHz and 1 MHz.

A calibration phantom was prepared to evaluate the accuracy of the LCR meter. The calibration phantom, as described previously,^74^ contained 0.15 M saline solution and was produced by dissolving 0.879 g of NaCl in 100 mL of water at room temperature. The conductivity value of the calibration phantom between 10 kHz and 1 MHz, as reported previously,^74^ is 1.39 S/m. The conductivity of the prepared phantom was also determined to be 1.40 S/m (Figure 3).

**FIGURE 4:**
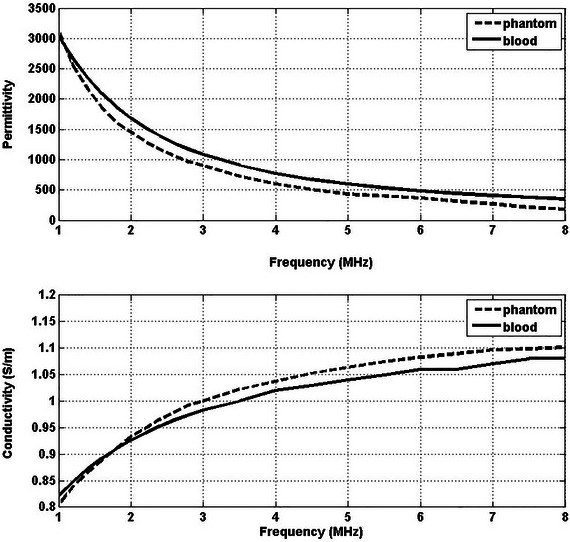
Conductivity and permittivity values of the phantom and blood (tumor) as functions of frequency.

**FIGURE 5:**
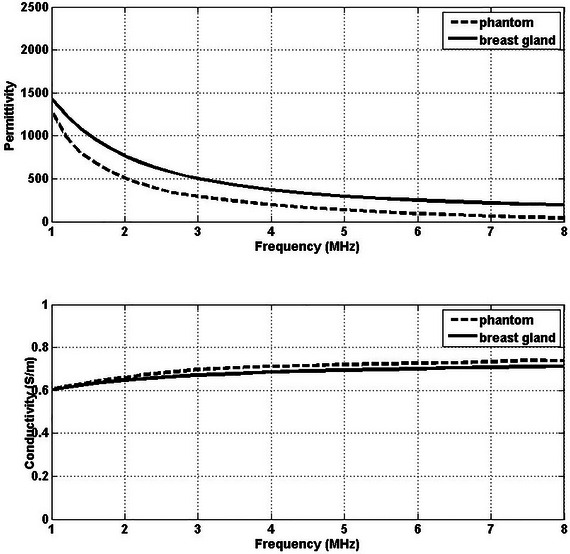
Conductivity and permittivity values of the phantom and breast gland as functions of frequency.

**FIGURE 6:**
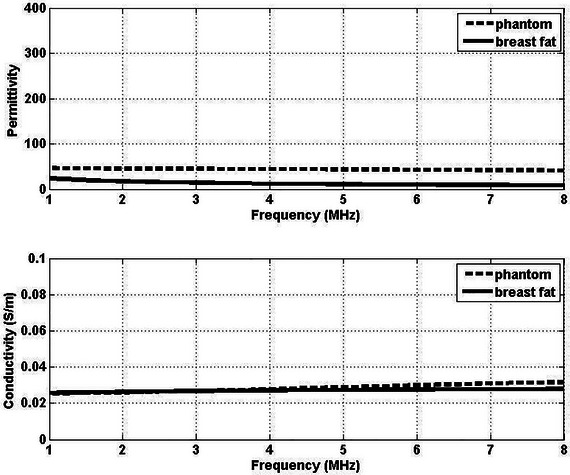
Conductivity and permittivity values of the phantom and breast fat as functions of frequency.

This study measured and averaged the electrical conductivity and dielectric constant values over five measurements. A comparison was made between the electric properties of the phantoms measured in this study and the tissue values reported in Ref. 75, as seen in Table 2. The measured conductivity and dielectric constant values of the phantoms are presented alongside the literature values in Figures 4, 5, 6.

**TABLE 2:** Measured electrical conductivity (Sm*^−^* ^1^) and relative permittivity (*ϵ_r_*) values compared with those reported in Ref. 75 for breast tissues at 1 MHz.

Tissues	Conductivity (Sm*^−^* ^1^)		Relative permittivity (*ϵ_r_*)	
	Tissues	Phantom	Tissues	Phantom
Breast gland	0.603	0.606±0.0023	1430	1288±8.0138
Breast fat	0.025	0.022±0.0016	23.7	49.02±0.6154
Tumor	0.822	0.813± 0.0033	3030	3000±17.5305

### Speed of sound results of phantoms

The experimental setups used to measure the ultrasonic speed of sound of the phantoms are depicted in Figure 2. The speed of sound in a phantom was measured via an ultrasonic transducer operating in amplitude mode. The phantom was placed in a glass container and then filled with water to submerge the phantom completely. Using an oscilloscope, we recorded two distinct voltage signals: the first from the boundary between the water and the phantom, and the second from the boundary between the phantom and the water. To determine the speed of sound within the phantom, we calculated the time difference between these two signals. This time interval corresponds to the duration of the sound wave to travel through the phantom. By knowing the depth of the phantom, *x*, and the time difference, *t*, we applied the formula *x* = *vt* to calculate the speed of sound, *v*, within the phantom.

The speed of sound of the prepared phantoms and the speed of sound of related tissues reported in the literature are presented in Table 3. This comparison was performed to assess the extent to which the developed phantoms accurately mimic the acoustic properties of biological tissues. These data play an important role in validating and developing the MAET technique.

**TABLE 3:** Measured speed of sound (ms*^−^* ^1^) values compared with those reported in Ref. 76 for breast tissues.

	Phantom (ms*^−^* ^1^)	Tissue (ms*^−^* ^1^)
Breast gland	1560	1430−1564
Breast fat	1227	1412−1490
Tumor	1660	1559−1590

## DISCUSSION

In our study, we prepared phantoms to mimic the electrical (conductivity and permittivity) and acoustic (speed of sound) properties of biological tissues, such as breast gland, breast fat, and tumor, to evaluate the breast tumor detection potential of MAET. The conductivity and relative permittivity values, as well as the sound velocities of these tissues, were measured and are presented in Tables 2 and 3. These measurements reveal clear differences between cancerous and healthy tissues, which could be used for imaging by MAET.

In comparing phantom models to real tissues, it was found that the phantoms effectively mimicked the electrical conductivity of their biological counterparts across various frequencies (1–8 MHz). Blood phantoms showed minimal differences (0.04−2.77%), breast fat phantoms exhibited variations (0.08−13.64%), and breast gland phantoms displayed manageable discrepancies (0.45−4.21%). These results highlight the potential of phantoms to closely mimic the conductivity characteristics of human tissues.

The phantom mimicking blood demonstrates a high degree of approximation of permittivity and conductivity across 1–8 MHz. This consistency renders it a valuable model for research involving blood tissue analogs. However, the phantom designed to mimic breast fat presents a considerable and persistent deviation in permittivity values, potentially diminishing its utility in scenarios where exact dielectric properties are imperative. In contrast, its mimicry of conductivity is closely aligned with that of actual breast fat, indicating potential relevance in studies prioritizing conductivity measurements. The breast gland phantom's performance is moderately satisfactory, especially at lower frequencies, though its precision in mimicking permittivity decreases at higher frequencies.

In the development of the breast fat phantom, the emphasis has been predominantly placed on accurately mimicking the conductivity characteristic of real breast fat tissue. This focused approach has provided valuable results regarding conductivity; however, the pronounced divergence in permittivity from the authentic tissue underscores an area for improvement. In the future, we aim to improve the permittivity values to more closely approximate those of real breast fat, developing a more accurate and comprehensive model for biomedical applications.

The ultrasonic sound velocities of the phantoms were also measured. Experimental setups were used to calculate the time it took for the sound to travel between different boundaries of the phantom. The calculated sound velocities were compared with the values in the literature. For the breast gland, the phantom has a speed of sound of 1560 m/s, whereas tissue values range from 1430 to 1564 m/s based on Ref. 76. This finding indicates that the phantom's speed of sound is within the range reported for breast gland tissues. The breast fat phantom has a speed of sound of 1227 m/s, which contrasts with tissue values ranging from 1412 to 1490 m/s. Here, the phantom's speed of sound falls below the reported range for breast fat tissues. The phantom exhibits a speed of sound of 1660 m/s for tumor tissue, compared with tissue values ranging from 1559 to 1590 m/s. The phantom's speed of sound is slightly higher than the upper limit of the reported range for tumor tissues.

In some studies, researchers have used phantoms made of agar and saline to mimic healthy tissues, but they have used fruits or vegetables to mimic malignant tissue for EIT systems. In the study by Holder et al.,^39^ cucumbers were utilized as phantoms to simulate malignant tissues. Similarly, Hong et al.^77^ used carrots to represent malignant tissues. To accurately mimic the dielectric constants of breast tissue, particularly at low frequencies (in this study, 1–8 MHz), we employed various chemical materials to closely mimic the dielectric constant values of breast fat, breast tissue, and tumor tissue. By facilitating the imitation of healthy and tumorous tissue properties, our study aims to increase the fidelity of models used in multifrequency EIT, especially MAET and other diagnostic modalities. In most previous studies, phantoms were generated to mimic the electrical properties of tissues, particularly breast tissues, in either the kHz range (up to 1 MHz) for EIT^78^, ^79^ or in the GHz range for microwave applications.^28^, ^29^

In a 2017 study,^30^ researchers developed a phantom designed to simulate healthy breast tissue using a gelatin‐agar mixture and cancerous tissue using a carrot. The study aimed to adjust the electrical properties of the phantoms by adding specific proportions of salt to the gelatin‐agar mixture and using a carrot to represent carcinoma tissue. Impedance spectra of the phantoms were measured across a 1–10 MHz frequency range and compared with measurements of healthy and carcinoma tissues reported in the literature. The results indicated that the impedance spectra of the phantoms show promise in mimicking carcinoma and healthy tissue. The impedance values of the carrot‐containing phantoms were higher than those of the gelatin‐agar phantoms mimicking healthy tissue. However, the impedance values of the gelatin‐agar mixture differed from those reported for real tissues in the literature, indicating that further optimization of the mechanical and electrical properties of the phantom is needed.

In some other studies,^80^, ^81^ researchers primarily aimed to measure and compare the dielectric properties of healthy and tumorous breast tissues, considering the classification of tissues based on fat content into high‐density, medium‐density, and low‐density tissues. Real breast tissue is heterogeneous, with fat content playing a critical role in determining the tissue's electrical properties and acoustic characteristics. High‐density tissues, which are characterized by low‐fat content (less than 20%) and high‐water content (over 70%), tend to have higher conductivity and permittivity values. These properties can enhance MAET's ability to detect small tumors and conductivity variations more easily in these tissues. In contrast, medium‐density tissues, which consist of a balanced proportion of fat and water (ranging from 20% to 80% fat), represent the most common breast tissue type.^80^, ^81^ The varying fat content in these tissues may present a mixed challenge for imaging techniques, and further evaluation using phantoms simulating medium‐density tissues would help better understand MAET's sensitivity to small conductivity changes in this category. Low‐density tissues, with high‐fat content (over 80%) and low‐water content (below 40%), generally have lower conductivity and permittivity values, which might reduce MAET's sensitivity in detecting small tumors or conductivity contrasts in these tissues.

Although our study utilizes a single fat content model, it is important to note that the varying electrical conductivities of low‐fat and high‐fat breast tissues can indeed influence MAET imaging outcomes. Relevant background on tissue composition is provided by two key studies: J. Kyber's 1992 paper^82^ and Gabriel's paper.^75^ Kyber's study used adipose tissue sourced from the peritoneal cavity of pigs, which contained approximately 97% fat, providing insights into high‐fat tissue characteristics. Gabriel's study, on the other hand, offered valuable data on the electrical properties of breast fat tissue, although it did not distinguish between low‐fat and high‐fat tissue types. These studies highlight the importance of fat content in the electrical properties of breast tissues, which may affect the sensitivity and accuracy of MAET in detecting small tumors.

While the use of a single phantom with a specific fat content was suitable for preliminary testing, we recognize that this limitation may not fully represent the broad spectrum of breast tissue densities encountered in clinical settings. To gain a more comprehensive understanding of how varying fat content influences MAET's performance, it would be highly beneficial to test additional phantom models that represent a wider range of fat densities. This would allow for a more robust evaluation of MAET's ability to detect small tumors and conductivity changes across different types of breast tissues, particularly in patients with high‐ or low‐fat content in their breast tissue. Future studies incorporating multiple phantom models with varying fat contents will significantly enhance our understanding of how breast density affects MAET imaging results, ultimately improving its accuracy and reliability in clinical applications.

In a review article by Zou and Guo in 2003,^83^ malignant breast tissues exhibit significantly lower electrical impedance (or higher conductivity) compared to surrounding healthy tissues. Specifically, malignant breast tumors have a higher conductivity at low frequencies (20 kHz to 100 MHz), ranging from 2.0–8.0 mS/cm, compared to 0.3–0.4 mS/cm in normal tissues. These electrical conductivity differences play a crucial role in distinguishing cancerous tissues from healthy ones, which MAET can detect with high sensitivity. MAET can detect conductivity differences as small as 0.1 S/m, which is sufficient to distinguish between cancerous and healthy tissues.^84^ For example, the conductivity of tumors is significantly higher than that of healthy mammary glands and adipose tissues—an important difference that can be detected using MAET.

Furthermore, the paper by Li and Liu^84^ also emphasizes the importance of acoustic impedance in distinguishing malignant tissues. Our measurements show that the speed of sound differs in tumors compared to surrounding healthy tissues, further enhancing MAET's ability to effectively distinguish these tissues. This finding supports the combination of both electrical conductivity and acoustic impedance contrasts in MAET for sensitive imaging. These results suggest that MAET has significant potential for clinical applications, particularly in the noninvasive detection of tumors. However, further clinical validation is required to optimize the methodology for use in patient studies.

In our study, we developed a homogeneous phantom for the initial stage of MAET. As of now, anisotropic phantom considerations have not been included in the MAET framework, primarily because MAET studies to date typically use simplified models to assess the feasibility and effectiveness of the technique. However, anisotropic phantoms have been employed in related imaging techniques such as EIT, which shares similar principles with MAET. For instance, Cao et al. developed an anisotropic phantom to model the acoustic properties of breast tissues, with direction‐dependent variations in sound propagation speed, which significantly improved the accuracy of ultrasonic imaging.^85^ Similarly, Sadleir et al. introduced an anisotropic model for EIT, considering direction‐dependent electrical conductivity, which enhanced the tissue characterization and tumor detection capabilities.^86^ In addition, Krzyzak et al.^87^ explored the use of anisotropic phantoms for magnetic resonance imaging. These studies highlight the potential benefits of incorporating anisotropic properties into phantom designs for more accurate imaging results. While these works are primarily based on EIT and related modalities, they indicate that anisotropic models could be essential for improving the accuracy of MAET in future studies, especially for deep tissue analysis and tumor detection.

## CONCLUSION

This study presents a novel approach to developing breast TM phantoms optimized for MAET experiments. Unlike previous studies that primarily focused on the electrical properties of breast phantoms at high frequencies, this work addresses both the electrical and acoustic characteristics within the 1–8 MHz range, aligning with the operational frequencies of ultrasound‐based diagnostic imaging. The phantoms, formulated using agar, sodium alginate, propanediol, aluminum powder, glycine, and graphite, successfully replicate the electrical conductivity, permittivity, and speed of sound of breast fat, glandular tissue, and tumors.

The measured electrical properties of the developed phantoms were compared with existing literature, demonstrating strong agreement with reported conductivity and permittivity values. This study also addresses a critical gap by incorporating both dielectric and acoustic parameters, essential for realistic tissue modeling.

Future research will focus on refining the phantom models to improve their permittivity accuracy, particularly for breast fat tissue, ensuring a closer match with biological tissues across a wider frequency range. Additionally, new phantom designs incorporating varying fat densities will be explored to better represent the heterogeneity of breast tissue and evaluate MAET's sensitivity across different tissue compositions. Further studies will also investigate the integration of MAET with complementary imaging modalities to enhance tumor detection accuracy. Finally, clinical validation studies will be conducted to assess the practical applicability of MAET in real‐world diagnostic settings.

## AUTHOR CONTRIBUTIONS

R.Z. and N.G.G. contributed equally to this manuscript.

## CONFLICT OF INTEREST STATEMENT

The authors do not have any conflicts of interest to disclose.

## Data Availability

The data supporting this study's findings are available upon request from the corresponding author.
